# Statistics of Cholera

**Published:** 1849-02

**Authors:** 


*Statistics of Cholera.*—For the following statistics of cholera, in other countries, quoted from various periodicals, we are chiefly indebted to the Medical News and Library. It will be perceived that the ratio of mortality in different countries, is remarkably uniform, being a little over one half the number of cases. The bearing of this fact upon the subject of treatment is deserving of notice.

In *Russia*, between the 28th of October, 1846, the period of the commencement of the epidemic, and the 5th of July, 1848, 290,318 persons were attacked, of whom 116,668 died.

*Berlin.*—From the 27th of July to the 11th of October, 1848, the numbers have been as follows: Number of cases, 2,009; deaths, 1,262; recoveries, 472; under treatment, 275.

*Hamburgh.*—The official report states that up to the 9th instant, the total number of persons attacked, was 2,229, of whom 1,043 had, up to that day, fallen victims; 411 remained under treatment, and 775 had been cured.

*Turkey.*—The accounts received from Constantinople by the last mail, announced that the cholera is gaining ground, the daily mortality averaging nearly 200.

At *Smyrna*, total attacked, 3,212. Total of deaths 2,494.

*Syria* and *Palestine.*—At Damascus, the deaths are reported as varying from 500 to 600 daily. Aleppo averags 150. Antioch about 50 daily.

*The Hague.*—In the cholera hospital 44 patients have been received; of these, 18 have died, 3 have recovered, and the remainder are under treatment.

*Scotland.*—Up to November 7, 468 cases had been reported; deaths, 243; recoveries, 54.—*Buffalo Medical Journal.*

